# Ability of naringenin, a bioflavonoid, to activate M-type potassium current in motor neuron-like cells and to increase BK_Ca_-channel activity in HEK293T cells transfected with α-*hSlo* subunit

**DOI:** 10.1186/s12868-014-0135-1

**Published:** 2014-12-24

**Authors:** Hung-Te Hsu, Yu-Ting Tseng, Yi-Ching Lo, Sheng-Nan Wu

**Affiliations:** Graduate Institute of Medicine, Kaohsiung Medical University, Kaohsiung, 80708 Taiwan; Department of Pharmacology, School of Medicine, Kaohsiung Medical University, Kaohsiung, 80708 Taiwan; Graduate Institute of Natural Products, School of Pharmacy, Kaohsiung Medical University, Kaohsiung, 80708 Taiwan; Department of Anesthesia, Kaohsiung Medical University Hospital, Kaohsiung City, 80708 Taiwan; Department of Physiology, National Cheng Kung University Medical College, Tainan City, 70101 Taiwan; Institute of Basic Medical Sciences, National Cheng Kung University Medical College, Tainan City, 70101 Taiwan

**Keywords:** Naringenin, M-type K^+^ channel, Large-conductance Ca^2+^-activated K^+^ channel, Motor neuron

## Abstract

**Background:**

Naringenin (NGEN) is a citrus bioflavonoid known to have beneficial health properties; however, the ionic mechanism of its actions remains largely unclear. In this study, we attempted to evaluate the possible effects of NGEN on K^+^ currents in NSC-34 neuronal cells and in HEK293T cells expressing α-*hSlo*.

**Results:**

NGEN increased M-type K^+^ current (*I*_K(M)_) in a concentration-dependent manner with an EC_50_ value of 9.8 μM in NSC-34 cells. NGEN shifted the activation curve of *I*_K(M)_ conductance to the more negative potentials. In cell-attached recordings, NGEN or flupirtine enhanced the activity of M-type K^+^ (K_M_) channels with no changes in single-channel amplitude. NGEN (10 μM) had minimal effect on *erg*-mediated K^+^ currents. Under cell-attached voltage-clamp recordings, NGEN decreased the frequency of spontaneous action currents and further application of linopirdine can reverse NGEN-induced inhibition of firing. In HEK293T cells expressing α-*hSlo*, this compound increased the amplitude of Ca^2+^-activated K^+^ current (*I*_K(Ca)_). Under inside-out recordings, NGEN applied to the intracellular side of the detached patch enhanced the activity of large-conductance Ca^2+^-activated K^+^ (BK_Ca_) channels. Moreover, from the study of a modeled neuron, burst firing of simulated action potentials (APs) was reduced in the presence of the increased conductances of both K_M_ and K_Ca_ channels. Fast-slow analysis of AP bursting from this model also revealed that as the conductances of both K_M_ and BK_Ca_ channels were increased by two-fold, the voltage nullcline was shifted in an upward direction accompanied by the compression of burst trajectory.

**Conclusions:**

The present results demonstrate that activation of both K_M_ and BK_Ca_ channels caused by NGEN might combine to influence neuronal activity if similar channels were functionally co-expressed in central neurons in vivo.

## Background

**Figure 1:**
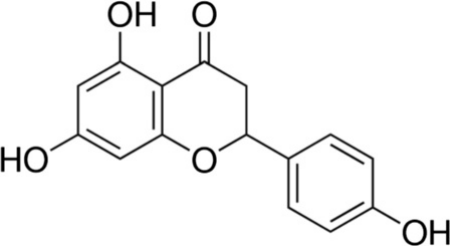
**Chemical structure of NGEN (naringenin, 4',5,7-trihydroxyflavanone).**

Naringenin (NGEN, Figure 1) is a major dietary flavonone, a type of flavonoid which is considered to have bioactive effects on human health. Several studies have indeed reported that it and other structurally related compounds could produce anxiolytic and antinociceptive actions [1-6]. An earlier study also demonstrated its ability to exert anti-inflammatory properties in macrophages and ex vivo human whole-blood models [7]. However, the ionic mechanism of their actions at the cellular level is largely unclear.

The KCNQ2, KCNQ3, and KCNQ5 genes are known to encode the core subunits of K_V_7.2, K_V_7.3 and K_V_7.5 channels. The increased activity of K_V_7.2, 7.3 and 7.5 channels is known to generate the M-type K^+^ current (*I*_K(M)_) which is a slowly activating and deactivating current suppressed by stimulation of muscarinic receptors [8]. Mutations of the KCNQ2 gene are notably involved in peripheral nerve hyperexcitability, a syndrome which is characterized by spontaneous and continuous muscle overactivity [9,10]. Most of *I*_K(M)_ in neurons are made by heterologously expressed K_V_7.2/7.3 channels [11,12]. Targeting K_V_7/K_M_ channels is recognized to be valuable as an adjunctive regimen for the treatment of many neurological disorders [8,13,14].

The large-conductance Ca^2+^-activated K^+^ channels (maxi-K channels, K_Ca_1.1, KCNMA1, *Slo*1) have the largest single-channel conductance of all K^+^ selective channels. They can be synergistically activated by membrane depolarization, elevation of intracellular Ca^2+^, or both. Native BK_Ca_ channels from mammalian tissues are composed of two structurally distinct subunits, α and β, arranged in a 1:1 stoichiometry. These channels are a physiologically and structurally diverse group of K^+^ channels that are essential for neuronal excitability by participating in the repolarization and after-hyperpolarization of action potentials [15,16].

NGEN has been previously shown to bind to GABA_A_ receptors and then to exert anxiolytic actions [1,2]. Earlier studies have demonstrated the ability of this agent to block HERG-encoded currents [17]. Another work also reported that it could dilate endothelium-denuded aortic ring by the activation of BK_Ca_ channels in vascular myocytes [18]. Naringin, another structurally similar compound, was recently reported to activate inwardly rectifying K^+^ channels [19]. Green tea flavonoids, such as epigallocatechin and epicatechin, were shown to reduce the activity of ATP-sensitive K^+^ channels [20]. Quercetin, another natural flavonoid, was described to alter the amplitude of L-type Ca^2+^ current in pituitary and motor neuron-like cells [21]. A recent report also showed the ability of NGEN to inhibit Cl^−^ secretion in isolated colonic epithelia [22]. Whether NGEN and its structurally related compounds can produce any effects on other types of ion channels including K_V_ channels remains incompletely understood.

The NSC-34 neuronal cell is a hybridoma cell line derived from the fusion of neuroblastoma cells with mice spinal cord cells. These cells have attracted growing interest as a suitable model for evaluation of the effects of potential neuroprotective compounds against different insults including excitotoxins, mitochondrial toxins and oxidants [23,24]. A recent study has reported the presence of Na^+^-activated K^+^ channels functionally expressed in these cells [25]. However, the biophysical or pharmacological properties in NSC-34 neuronal cells are incompletely characterized.

In the current study, we provide the first evidence that NGEN can interact with K_M_ channels to increase the amplitude of macroscopic *I*_K(M)_ in motor neuron-like NSC-34 cells. In HEK293T cells transfected with α-*hSlo* subunit, NGEN was also noted to increase the activity of BK_Ca_ channels. Therefore, similar to BMS-204352-induced change in BK_Ca_ and neuronal KCNQ channels [13,26], both stimulation of *I*_K(M)_ and *I*_K(Ca)_ caused by NGEN can be a potential mechanisms through which it influences neuronal activity in central neurons, although these two compounds are structurally distinguishable. Furthermore, numerical simulation of AP bursting generated from a modeled pyramidal neurons [27] clearly showed that as the conductances of g_M_ and g_KCa_ channels were elevated by two-fold to mimic the stimulatory actions of NGEN, the intraburst firing of APs was reduced accompanied by the increased after-hyperpolarization.

## Results

### Effect of NGEN on I_K(M)_ in NSC-34 cells

**Figure 2:**
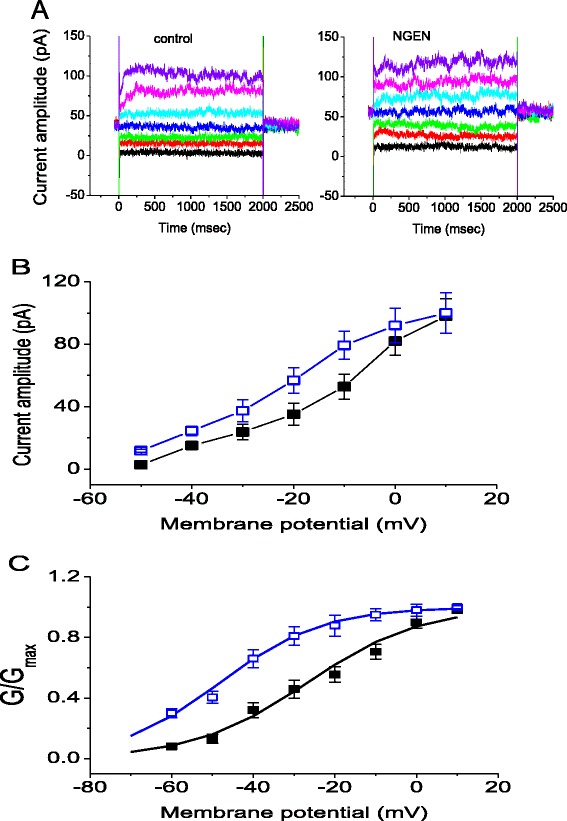
**Effects of NGEN on** ***I***_**K(M)**_ **recorded from motor neuron-like NSC-34 cells.** In **(A)**, superimposed current traces obtained in the absence (left) and presence (right) of 10 μM NGEN. In these experiments, cells were bathed Ca^2+^-Tyrode’s solution which contained 200 nM iberiotoxin, 200 nM apamin and 1 μM tetrodotoxin. The *I*_K(M)_ was elicited from -20 mV to different potentials which ranged from -50 to +10 mV with 10-mV increments. **(B)** Effect of NGEN on the averaged *I-V* relations of *I*_K(M)_ in NSC-34 cells (mean ± SEM; n = 9-13 for each point). ■: control; □: 10 μM NGEN. Current amplitude was measured at end of each voltage pulse. **(C)** Voltage dependence of *I*_K(M)_ conductance in the absence (■) and presence (□) of 10 μM NGEN (mean ± SEM; n = 8-12 for each point). Note that there is a leftward shift in the activation curve of *I*_K(M)_ conductance during cell exposure to NGEN, although the slope factor remains unchanged.

In these experiments, NSC-34 cells were bathed in Ca^2+^-free Tyrode’s solution. To ensure that the activity of K_Ca_ or K_Na_ channels was not contaminated, iberiotoxin (200 nM), apamin (200 nM) and tetrodotoxin (1 μM) were added to the bath medium. Iberiotoxin and apamin are, respectively, the blockers of BK channels and small-conductance Ca^2+^-activated K^+^ channels, while tetrotodotxin is a potent blocker of voltage-gated Na^+^ current. Under whole-cell configuration, the *I*_K(M)_ was elicited from a holding potential of -20 mV to different potentials which ranged from -50 to +10 mV with 10-mV increments at a rate of 0.05 Hz. The voltage protocol used was previously described [28]. As shown in Figure 2, addition of NGEN at a concentration of 10 μM increased the amplitude of *I*_K(M)_ throughout the entire voltage-clamp steps examined. For example, at the level of -10 mV, cell exposure to NGEN (10 μM) significantly elevated *I*_K(M)_ amplitude from 52.9 ± 8 to 79.2 ± 9 pA (n = 11). After washout of NGEN, *I*_K(M)_ amplitude returned to 57 ± 8 pA (n = 7). Similarly, flupirtine (10 μM), an activator of *I*_K(M)_ [8,14], also increased the *I*_K(M)_ amplitude found in NSC-34 cells. A further application of linopirdine (10 μM) reversed *I*_K(M)_ amplitude to 55.6 ± 6 pA observed at the same level of holding potential. Linopirdine is a blocker of *I*_K(M)_. Figure 2B illustrates averaged *I-V* relationships of *I*_K(M)_ obtained in the control and during exposure to NGEN. Specifically, cell exposure to NGEN significantly increased the slope of the linear fit of *I*_K(M)_ amplitudes to the voltages between -30 and -10 mV, namely, whole-cell conductance of *I*_K(M)_, from 1.45 ± 0.03 to 2.11 ± 0.05 nS (n = 9). The data clearly indicate that as NSC-34 cells are exposed to NGEN, the *I-V* relationship of *I*_K(M)_ in NSC-34 cells can be modified.

### Effect of NGEN on the voltage dependence of I_K(M)_ activation in NSC-34 cells

Figure 2C shows the activation curve of *I*_K(M)_ obtained with or without addition of NGEN (10 μM). The plot of relative *I*_K(M)_ conductance as a function of membrane potential was constructed and fitted with a Boltzmann function as described under Methods. In controls, V_1/2_ = -26.8 ± 0.7 mV, *q* = 1.86 ± 0.07 *e* (n = 9), whereas in the presence of NGEN (10 μM), V_1/2_ = -44.3 ± 1.1 mV and *q* = 2.01 ± 0.06 *e* (n = 7). The data showed that the activation curve of *I*_K(M)_ was shifted along the voltage axis to more negative potentials by approximately 18 mV, as NSC-34 cells were exposed to NGEN. However, no significant change in the gating charge was clearly demonstrated in the presence of NGEN. The data thus indicate that in NSC-34 cells, NGEN is capable of shifting the activation curve of *I*_K(M)_ to more negative potentials with no discernible change in the apparent gating charge.

**Figure 3:**
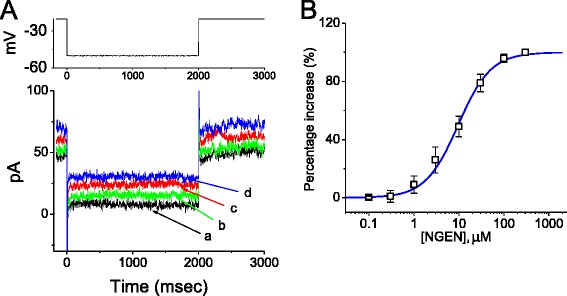
**Concentration-dependent stimulation of** ***I***_**K(M)**_ **by NGEN in NSC-34 cells.** In these experiments, each cell was hyperpolarized from -20 to -50 mV with a duration of 2 sec. **(A)** Original current traces obtained in the control and during exposure to NGEN. a: control; b: 3 μM NGEN; c: 10 μM NGEN; d: 30 μM NGEN. The upper part indicates the voltage protocol used. **(B)** Concentration-response relationship for NGEN-induced increase of *I*_K(M)_ (i.e., linopirdine-sensitive current) measured at the end of hyperpolarizing pulses (mean ± SEM; n = 9-12 for each point). Smooth blue line represents the best fit to the Hill equation as described in Methods.

The relationship between the NGEN concentration and the percentage increase of *I*_K(M)_ is illustrated in Figure 3. To evoked *I*_K(M)_ obtained in controls and during exposure to different concentrations (0.1-300 μM) of NGN, each cell was hyperpolarized from -20 to -50 mV. The amplitude of *I*_K(M)_ measured at the end of hyperpolarizing pulse was compared with those obtained after subsequent application of linopirdine (30 μM). Addition of NGEN (0.1-300 μM) was noted to increase the amplitude of linopirdine-sensitive current in a concentration-dependent manner. The half-maximal concentration (i.e., EC_50_) required for stimulatory effect of NGEN on *I*_K(M)_ was calculated to be 9.8 ± 0.4 μM, and at a concentration of 300 μM, it increased almost all of the *I*_K(M)_ amplitude in these cells.

### Effects of various related compounds on I_K(M)_ in NSC-34 cells

**Figure 4:**
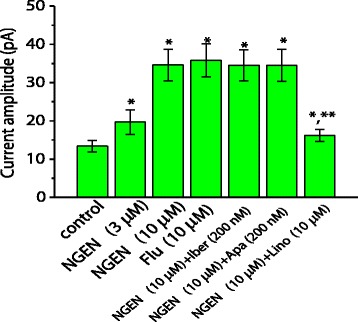
**Effects of NGEN and other K** ^**+**^ **current blockers on the amplitude of** ***I***_**K(M)**_ **in NSC-34 cells.** In these experiments, cells were bathed in Ca^2+^-free Tyrode's solution which contained 200 nM iberiotoxin, 200 nM apamin and 1 μM tetrodotoxin, and each cell was hyperpolarized from -20 to -50 mV with a duration of 2 sec. Current amplitude was measured at the end of voltage pulse. Each bar represents the mean ± SEM (n = 7-13). Flu: flupirtine; Iber: iberiotoxin; Aps: apamin; Lino: linopirdine. ^*^Significantly different from control. ^**^Significantly different from NGEN (10 μM) alone group. Notably, further application of linopirdine reversed NGEN-stimulated *I*_K(M)_, while neither iberiotoxin nor apamin produced any effects on it.

Earlier reports have shown the ability of NGEN or its structurally similar compounds to alter K^+^-channel activity [18,19]. Effects of NGEN, flupirtine, NGEN plus linopirdine and NGEN plus blocker of *I*_K(Ca)_ on *I*_K(M)_ were further examined and compared. As shown in Figure 4, similar to NGEN, flupirtine at a concentration of 10 μM was effective in increasing the amplitude of *I*_K(M)_. Flupirtine is recognized to be a specific activator of neuronal KCNQ channels [14]. However, neither iberiotoxin nor apamin produced any effects on NGEN-stimulated *I*_K(M)_, although subsequent application of linopirdine was able to reverse the increased *I*_K(M)_ caused by either NGEN or flupirtine. Therefore, NGEN-stimulated *I*_K(M)_ is subject to inhibition by linopirdine, but not linked to the activity of K_Ca_ channels probably expressed in NSC-34 cells,

### Effect of NGEN on K_M_ channels in NSC-34 neuronal cells

**Figure 5:**
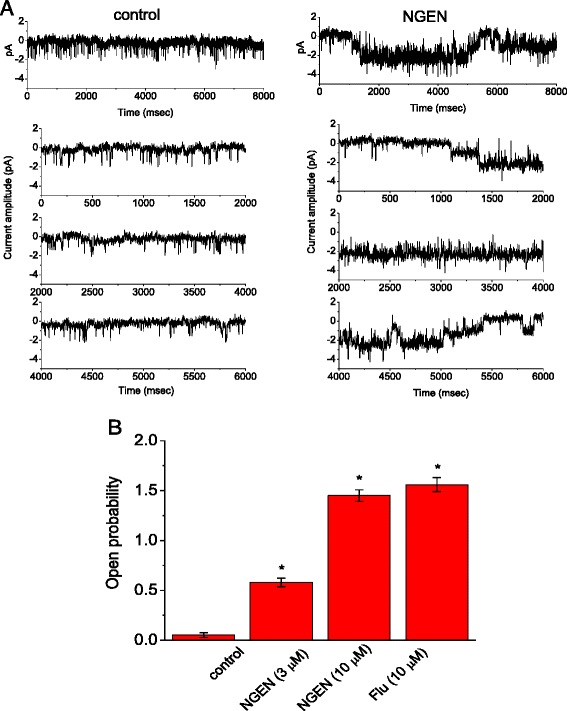
**Stimulatory effect of NGEN on the activity of K**_**M**_ **channels recorded from NSC-34 cells.** In **(A)**, cells were bathed in Ca^2+^-free Tyrode’s solution which contained iberiotoxin (200 nM), apamin (200 nM) and tetrodotoxin (1 μM). Cell-attached configuration was made as the cell attached was held at 0 mV relative to the bath. Channel activity was obtained in the control (left) and after addition of NGEN (10 μM) to the bath. Portion of tracing in the upper part of **(A)** is amplified in the lower part. Channel openings give a downward deflection in current. **(B)** Summary of the data showing effects of NGEN and flupirtine (Flu) on K_M_-channel activity in NSC-34 cells (mean ± SEM; n = 9-12 for each bar). ^*^Significantly different from control.

We next sought to investigate whether NGEN can act by influencing the activity of K_M_ channels for changes in whole-cell *I*_K(M)_ amplitude in these cells. In these experiments, cell-attached recordings were conducted in cells bathed in Ca^2+^-free Tyrode’s solution. To ensure that the activity of K_Ca_ or K_Na_ channels would not be contaminated, iberiotoxin (200 nM), apamin (200 nM) and tetrodotoxin (1 μM) were added to the bathing solution. The recording pipette was filled with K^+^-containing solution and the cell attached to the pipette was held at 0 mV relative to the bath. As the cell was in a physiological external solution, the resting potential of the cells was about -71 mV. The pipette solution had a high K^+^ concentration, which is the same as that of the cytoplasm. Therefore, a K^+^ channel in the membrane patch would be expected to have a reversal potential of 0 mV. The membrane patch can be depolarized by applying negative command potentials to 0 mV. Figure 5A shows the current tracings of single-channel recordings obtained with or without addition of NGEN (10 μM). When NGEN was added to the bath, channel activity was greatly raised. The probability of channel openings was significantly elevated from 0.052 ± 0.024 to 1.45 ± 0.058 (n = 9) in the presence of 10 μM NGEN (Figure 5B). Similarly, flupirtine (10 μM) was effective in increasing channel activity. A further application of 2 mM CaCl_2_ or linopirdine (10 μM) to the bath could effectively reverse NGEN-stimulated activity of K_M_ channels. However, single-channel amplitude between the absence and presence of NGEN or flupirtine did not differ significantly.

### Inability of NGEN to block I_K(erg)_ in NSC-34 neuronal cells

**Figure 6:**
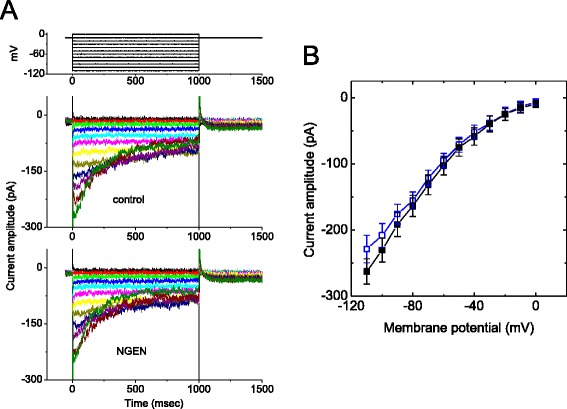
**No effects of NGEN on** ***I***_**K(erg)**_ **in NSC-34 cells.** In these experiments, cells were bathed in a high-K^+^, Ca^2+^-free solution. Each cell was held at -10 mV and various voltage pulses ranging from -110 to 0 mV with 10-mV increments were applied as shown in the uppermost part of **(A)**. **(A)** Superimposed *I*_K(erg)_ obtained in the absence (upper) and presence (lower) of 10 μM NGEN. **(B)** Averaged *I-V* relationship of peak *I*_K(erg)_ obtained in control (■) and during exposure to 10 μM NGEN (□) (mean ± SEM; n = 8-13 for each point).

Earlier work has demonstrated the ability of NGEN to block cardiac HERG currents [17]. We further investigated the possible effects of NGEN on *I*_K(erg)_ found in NSC-34 cells. As depicted in Figure 6, addition of NGEN at a concentration of 10 μM did not cause any effect on peak *I*_K(erg)_ amplitude elicited throughout the entire voltage-clamp steps examined. The peak amplitude of *I*_K(erg)_ elicited by membrane hyperpolarizations was not noted to differ significantly between the absence and presence of 10 μM NGEN. However, addition of azimilide, an blocker of *I*_K(erg)_ [29], reduced *I*_K(erg)_ amplitude by 43%. The results presented here are compatible with previous observations showing that the IC_50_ value for NGEN-induced block of HERG channels expressed in *Xenopus* oocytes is about 100 μM [17], which is much greater than that used to stimulate *I*_K(M)_ described here.

### Effects of NGEN on spontaneous action currents recorded from NSC-34 cells

**Figure 7:**
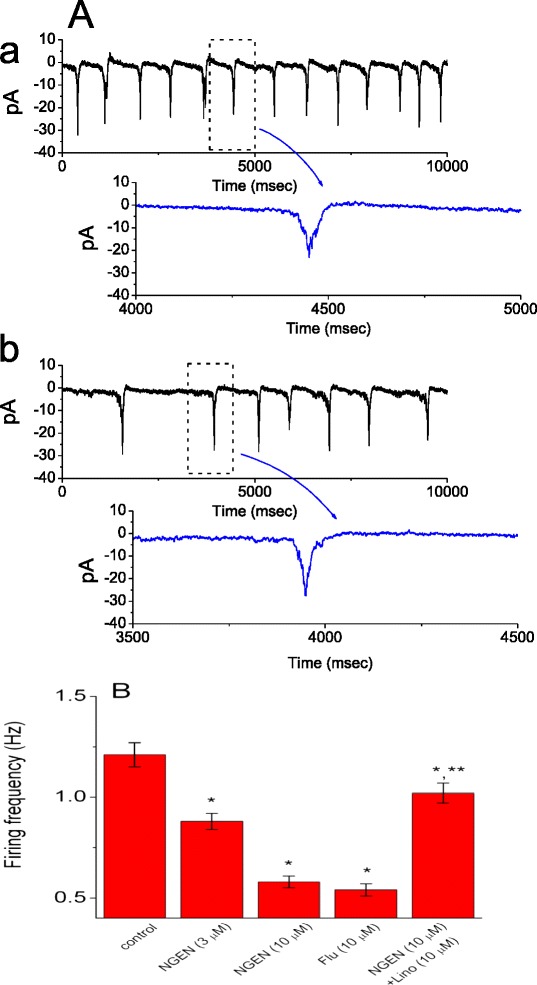
**Effects of NGEN and flupirtine on spontaneous action currents recorded from NSC-34 neuronal cells.** In **(A)**, current traces were obtained in the absence (*a*) and presence (*b*) of 10 μM NGEN. The lower part shown at (*a*) and (*b*) indicates an expanded record from dashed box. Notably, the traces showing downward deflections indicate action currents. **(B)** Summary of the data showing effects of NGEN, flupirtine (Flu), NGEN plus linopirdine (Lino) on the frequency of action currents in NSC-34 cells. Each point indicates the mean ± SEM (n = 7-10). ^*^Significantly different from control.

It was also examined whether NGEN can produce any changes in spontaneous action currents emerging in these cells. In these experiments, cells were bathed in normal Tyrode's solution containing 1.8 mM CaCl_2_. Cell-attached voltage-clamp recordings were performed [30,31] and patch pipettes were filled with a K^+^-containing solution. The potential across the patch was set at the level of the resting membrane potential of the cells (around -70 mV). As illustrated in Figure 7, addition of NGEN caused a significant reduction in the firing of spontaneous action currents. The frequency of action currents in the presence of 10 μM NGEN (0.54 ± 0.03 Hz, n = 7) was significantly smaller than that in control (1.21 ± 0.06 Hz, n = 8). Moreover, a subsequent application of linopirdine (10 μM) reversed the firing frequency to 1.02 ± 0.05 Hz (n = 6), although iberiotoxin (200 nM) had minimal effects on NGEN-induced decrease of firing frequency. In light of these data, we assumed that NGEN-induced reduction of firing frequency is primarily linked to the simulation of K_M_ channels and unlikely to be mediated through the activation of BK_Ca_ channels.

### Stimulatory effect of NGEN on I_K(Ca)_ in HEK293T cells transfected with α-hSlo

**Figure 8:**
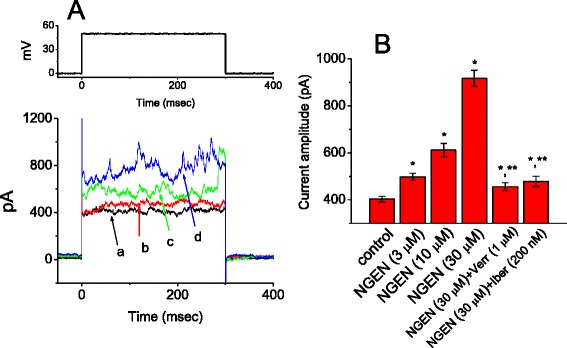
**Effect of NGEN on whole-cell** ***I***_**K(Ca)**_ **in α** ***-hSlo*** **-expressing HEK293T cells.** In these experiments, cells were bathed in normal Tyrode’s solution containing 1.8 mM CaCl_2_. **(A)** Current traces in response to membrane depolarization from 0 to +50 mV. The upper part indicates the voltage protocol used. a: control; b: 3 μM NGEN; c: 10 μM NGEN; and d: 30 μM NGEN. **(B)** Summary of the data showing effects of NGEN, NGEN plus verruculogen (Verr; 1 μM) and NGEN plus iberiotoxin (Iber; 200 nM) on *I*_K(Ca)_ amplitude in these cells (mean ± SEM; n = 10-13 for each bar). ^*^Significantly different from control. ^**^Significantly different from NGEN (30 μM) alone group.

Previous work has demonstrated the ability of NGEN to stimulate the activity of BK_Ca_ channels in vascular smooth myocytes [18]. We also tested the hypothesis that this compound exerts any effects on *I*_K(Ca)_ in HEK293T cells expressing α-*hSlo*. Under our experimental conditions [32], transfection with α-*hSlo* into HEK293T cells can result in the appearance of BK_Ca_ channels, thereby elevating the amplitude of macroscopic *I*_K(Ca)_. In whole-cell configuration, as NGEN was applied to the bath, the *I*_K(Ca)_ amplitude was significantly increased (Figure 8). For example, NGEN at a concentration of 30 μM increased *I*_K(Ca)_ amplitude at +50 mV from 403 ± 12 to 917 ± 34 pA (n = 11). Subsequent application of verruculogen (1 μM) or iberiotoxin (200 nM) was noted to reverse NGEN-stimulated *I*_K(Ca)_ significantly (Figure 8B); however, neither apamin (200 nM) nor linopirdine (10 μM) had any effects on it.

### Stimulatory effect of NGEN on the activity of BK_Ca_ channels in HEK293T cells expressing α-hSlo

**Figure 9:**
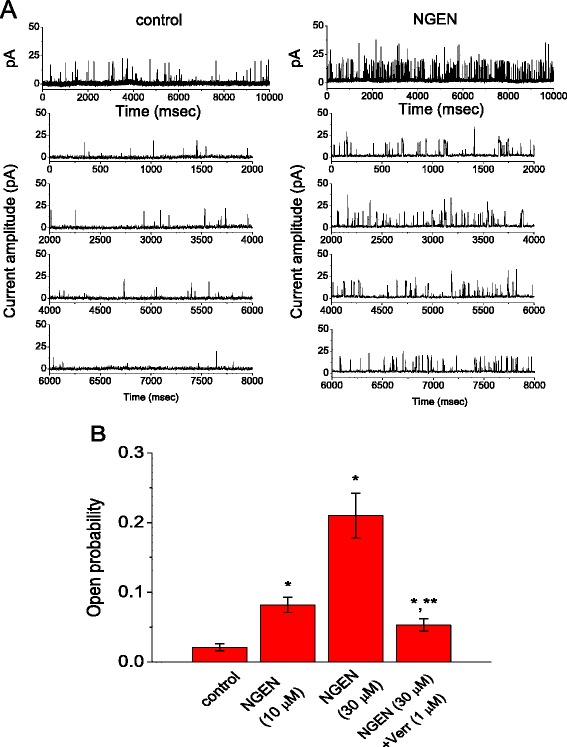
**Stimulatory effect of NGEN on BK**_**Ca**_ **-channel activity measured from α** ***-hSlo*** **-expressing HEK293T cells.** **(A)** Original current traces showing the activity of BK_Ca_ channels before (left) and after application (right) of 30 μM NGEN. Inside-out recordings were conducted with symmetrical K^+^ concentration (145 mM). The potential was constantly held at +60 mV, and bath medium contained 0.1 μM Ca^2+^. Channel openings give an upward deflection in current. **(B)** Summary of the data showing effect of NGEN and NGEN plus verruculogen (Verr; 1 μM) on the probability of BK_Ca_-channel openings (mean ± SEM; n = 8-11 for each bar). ^*^Significantly different from control. ^**^Significantly different from NGEN (30 μM) alone group.

Verruculogen and iberiotoxin, known inhibitors of BK_Ca_ channels [33], could clearly reverse NGEN-stimulated *I*_K(Ca)_ in these cells. The increased macroscopic *I*_K(Ca)_ caused by NGEN could be due to either the elevated open probability, an increase in the number of active channels, or both. The effect of NGEN on BK_Ca_-channel activity was further investigated (Figure 9). In this set of experiments, single-channel recordings with an inside-out configuration were performed in symmetrical K^+^ solution (145 mM). Bath medium contained 0.1 μM Ca^2+^ and the potential was held at +60 mV. When NGEN (30 μM) was applied to the intracellular leaflet of the detached patch, channel activity was significantly raised to 0.211 ± 0.032 from a control of 0.021 ± 0.005 (n = 9). A further application of verruculogen (1 μM) decreased the probability of channel openings to 0.053 ± 0.009 (n = 8), although linopirdine (10 μM) applied to the bath had minimal effects on NGEN-stimulated channel activity. The single-channel conductance of BK_Ca_ channels between the absence and presence of 30 μM NGEN was not noted to differ significantly (178 ± 6 pS [control] versus 180 ± 6 pS [NGEN], n = 7). Therefore, in agreement with previous observations made in vascular myocytes [18], the NGEN-induced increase of *I*_K(Ca)_ amplitude in HEK293T cells which were transfected with α-*hSlo*, tended to be associated with its increase in the probability of channel openings, rather than changes in the number of functional active channels. NGEN can thus interact with BK_Ca_ channels to increase the amplitude of *I*_K(Ca)_ in these cells, even in the absence of BK_Ca_-channel β-subunits.

### Simulated firing and bursting pattern of APs in central modeled neuron with varying g_M_ or g_KCa_

**Figure 10:**
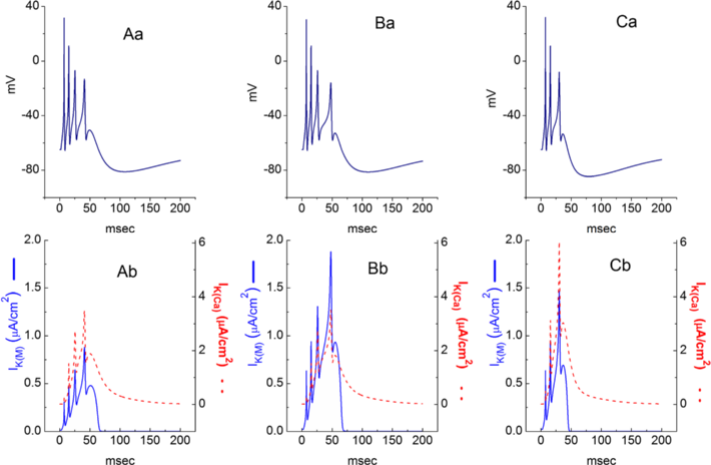
**Burst firing of APs (upper parts) and the corresponding changes of** ***I***_**K(M)**_ **and** ***I***_**K(Ca)**_ **(lower parts) in modeled hippocampal neuron.** The detailed formulations were described under Methods. The firing was generated with the values of g_M_ and g_KCa_ was set at 0.02 and 0.05 mS/cm^2^ **(Aa)**, 0.04 and 0.05 mS/cm^2^ **(Ba)** and 0.04 and 0.1 mS/cm^2^ **(Ca)**, respectively. Ab, Bb, and Cb indicate the corresponding changes in *I*_K(M)_ (blue solid lines) and *I*_K(Ca)_ (red dashed lines), respectively. In **(C)**, the values of g_M_ and g_KCa_ were arbitrarily elevated by two-fold to mimic the stimulatory effects of NGEN (10 μM). Note that in this simulation, the amplitudes of *I*_K(M)_ are smaller than those of *I*_K(Ca)_.

We further explored how the dynamics of bursting firing elicited by continuous current injection can be altered by increasing the values of g_M_ and g_KCa_ to mimic the stimulatory of NGEN on *I*_K(M)_ and *I*_K(Ca)_ described above. For studying this, a brief depolarizing current with 0.004 mA was applied to the modeled central neuron (e.g., CA3 pyramidal neuron) with the parameter values illustrated in Table 1, in an attempt to generate burst firing of neuronal APs. The descriptions for this modeled neuron were detailed under Methods. Initially, the g_M_ value was arbitrarily elevated from 0.02 to 0.04 mS/cm^2^. Subsequently, to mimic the condition in which NGEN (10 μM) was added, the values of g_M_ and g_KCa_ was arbitrarily raised by two-fold. As illustrated in Figure 10, by elevating g_M_ value alone, we are able to show an increase in the intra-burst interval in combination with the increased *I*_K(M)_. As both values of g_M_ and g_KCa_ were elevated, intra-burst rate was reduced, along with the increase of after-hyperpolarizaton from -81 to -85 mV (Figure 10Ca). It also needs to be noted that in Figure 10Cb, the *I*_K(M)_ amplitude numerically generated was increased only by 1.5-fold, although g_M_ value was elevated by 2-fold as compared with the results in Figure 10Bb. Therefore, it is possible from these simulation results that when both g_M_ and g_KCa_ are at work simultaneously, the outcome may not simply be due to the summation of individual element.

**Table 1:** **Default parametric values used for the modeling of hippocampal CA3 pyramidal neurons**

**Symbol**	**Description**	**Value**
Cm	Membrane capacitance (pF)	1
g_Na_	Na^+^ current conductance (S/cm^2^)	2
g_CaT_	T-type Ca^2+^ current conductance (S/cm^2^)	0.45
g_CaL_	L-type Ca^2+^ current conductance (S/cm^2^)	0.0025
g_CaN_	N-type Ca^2+^ current conductance (S/cm^2^)	0.0025
g_KDR_	Delayed rectifier K^+^ current conductance (S/cm^2^)	0.08
g_A_	A-type K^+^ current conductance (mS/cm^2^)	0.1
g_M_	M-type K^+^ current conductance (mS/cm^2^)	0.02
g_KCa_	Ca^2+^-activated K^+^ current conductance (mS/cm^2^)	0.05
g_Kahp_	After-hyperpolarization K^+^ current conductance (mS/cm^2^)	0.0018
g_leak_	Leak current conductance (mS/cm^2^)	0.0167
I_app_	Applied current (mA)	0.004
V_Na_	Na^+^ reversal potential (mV)	50
V_K_	K^+^ reversal potential (mV)	−91
V_Leak_	Reversal potential for leak current (mV)	−65

### Fast-slow analysis of AP bursting in model neuron

Finally, in order to develop a better quantitative understanding of K_M_ and K_Ca_ channels, we further performed fast-slow analysis in a reduced comparable model. Such a maneuver can be allowed for separation of the variables into two subsystems, i.e., fast (fully responsive within the timescale of a spike) and slow (changes on the timescale of the whole burst) [27,34]. One can analyze how the bursting from this modeled neuron is numerically generated owing to a delicate interaction of these two variables. In other words, one is a slow autocatalytic variable **n** (i.e., activation gate for Ca_V_3.1 T-type Ca^2+^ channel) and the other is a slow negative feedback variable **o** (i.e., activation gate for K_Ca_ channel).

**Figure 11:**
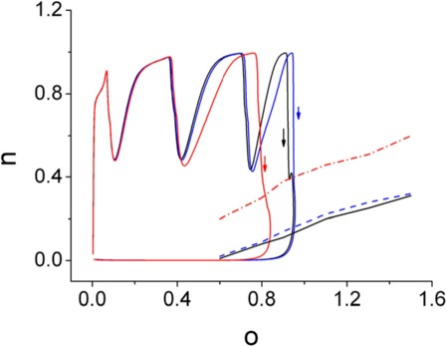
**Fast-slow analysis of AP bursting generated from modeled neuron.** The phase plot of burst firing elicited by continuous current injection shows the projection of the burst trajectory onto **n-o** plane. **n** and **o** denote the activation gates of Ca_V_3.1 and K_Ca_ channels, respectively. Arrows indicate the direction of trajectory movement. The solid and dashed lines are the voltage nullcline (i.e., d*V*/d*t* = 0) of the fast subsystem with **n** and **o** as parameters. Below the nullcline is the silent region. Notably, as the value of g_M_ was increased from 0.02 to 0.04 mS/cm^2^, the nullcline was shifted in an upward direction with minimal change in the burst trajectory. When the values of g_M_ and g_KCa_ were simultaneously elevated by two-fold to mimic the effect of NGEG (10 μM), the burst trajectory was clearly compressed in combination with a further upward shift in the voltage nullcline.

In this analysis, we treated slow variables **n** and **o** as the parameters to study the fast subsystem and subsequently to determine the regions of spiking and resting states, as the different values of g_M_ and g_KCa_ are chosen. As shown in Figure 10, the two-parameter phase plot clearly showed the ability of the burst trajectory to be projected onto the slow variables (i.e., **n-o** plane). The solid and dashed lines shown in Figure 11, correspond to the voltage nullcline (d*V*/d*t* = 0) for the fast system at the indicated values of **n** and **o**, where the saddle node bifurcation occurs. It becomes clear from this plot that an interaction of **n** and **o** was able to generate slow oscillations that were allowed to move the fast subsystem in and out of the repetitive spiking regime. The arrows shown in Figure 10 indicate the direction of the trajectory movement. The region above the nullcline corresponds to a region of repetitive firing, while the region below the nullcline is where the system remains silent. The arbitrary increase of g_M_ value from 0.02 to 0.04 mS/cm^2^ was noted to shift voltage nullcline in an upward direction with minimal changes in burst trajectory on **n-o** plane. However, of interest, when the values of g_M_ and g_KCa_ were simultaneously elevated by two-fold to mimic the stimulatory actions of NGEN (10 μM) described above, the nullcline was further shifted in an upward direction accompanied by the compression of burst trajectory in size on **n-o** plane. Such upward shift in the voltage nullcline indicates a reduction of repetitive spiking regime. The results of these changes are therefore compatible with our experimental and computational data showing that the increased values of g_M_ and g_KCa_ caused by NGEN result in a reduction of intraburst firing accompanied by the prolongation of interburst period and the increase of the afterhyperpolarization of APs, if similar findings are observed in central neurons in vivo.

## Discussion

In this study, we provide the direct evidence that NGEN has a stimulatory action on *I*_K(M)_ in NSC motor neuron-like cells. The half-maximal concentration (i.e., EC_50_) of NGEN required for stimulation of *I*_K(M)_ was about 9.8 μM, which is much smaller than that reported for the inhibition of HERG channels [17]. Because of their lipophilicity, NGEN was also reported to transverse the blood-brain barrier and subsequently to penetrate into different brain regions [35], although the brain concentrations of NGEN vary based on local extracellular milieu in and around membranes and synapses. The importance of K_V_7 channels as regulators of neuronal excitability became apparent, when mutated neuronal K_V_7 channels were demonstrated to cause benign familial neonatal convulsions [9]. It is thus anticipated that in motor neurons like NSC-34 cells, the K_V_7/K_M_ channels (i.e., KCNQ2/Q3 and/or KCNQ3/Q5 heteromultimers) are an important therapeutic target for the action of this agent or other structurally similar compounds within the CNS.

Addition of NGEN could shift the activation curve of *I*_K(M)_ conductance to the hyperpolarized potential in this study. Inability of NGEN to alter the gating charge of K_M_ channels observed in NSC-34 cells allows us to suggest that the stimulatory effect of this compound on K_M_ channels in NSC-34 cells is not mediated through a direct effect on the voltage sensor per se. The binding site of NGEN is most likely to lie outside of the transmembrane field around the channel. Nevertheless, as the neurons are exposed to NGEN, the effects of this compound on *I*_K(M)_ amplitude could rely upon the NGEN concentration used, the level of pre-existing membrane potential, or both.

The pharmacological and electrophysiological properties of K_M_ channels observed in NSC-34 cells are similar to those in other types of neurons described previously [8,11,36]. In cell-attached configuration, addition of NGEN (10 μM) into the bath was noted to increase the probability of channel openings with no discernible change in single-channel amplitude. The inability of NGEN to modify single-channel conductance indicates that the increased responsiveness of the channel to this compound is not located at the central ion-conducting pore of the channel. Similarly, flupirtine at a concentration of 10 μM was capable of enhancing K_M_-channel activity in these cells. The stimulatory actions of NGEN and flupirtine on the activity of K_M_ channels were not additive in NSC-34 neuronal cells (data not shown). Our results suggest that the two compounds exert their major effects on the same component existing in K_M_ channels. Further work is also required to see whether this compound can interact with the K_V_7/K_M_ (KCNQ2-7) channels to stimulate *I*_K(M)_ in other types of central neurons.

Previous reports have demonstrated the ability of NGEN to bind to GABA_A_ receptors and to produce anxiolytic actions [1,2]. A recent study also showed that NGEN might inhibit Cl^−^ secretion in isolated colonic epithelia [22]. In our study, macroscopic *I*_K(M)_ was measured in the recording pipette which was filled with a solution containing aspartic acid, rather than Cl- ions. Moreover, gabazine (10 μM), a blocker of GABA_A_ receptors, had minimal effects on NGEN-stimulated *I*_K(M)_ in NSC-34 cells. It thus seems unlikely that the observed increase of *I*_K(M)_ caused by NGEN is inherently associated with the increase of Cl^−^ current induced by its binding to GABA_A_ receptors.

An earlier work has demonstrated that green tea flavonoids such as epigallocatechin and epicatechin were capable of reducing the activity of ATP-sensitive K^+^ channels [20]. Under our experimental condition, the recording pipette used for measurements of whole-cell *I*_K(M)_ was filled with a solution which contained 3 mM ATP. Moreover, NGEN-stimulated *I*_K(M)_ observed in NSC-34 cells was not reversed by subsequent application of glibenclamide (30 μM), but by linopirdine (10 μM) (data not shown). Therefore, in NSC-34 cells or in HEK293T cells expressing α-*hSlo*, the elevation of K^+^ currents induced by NGEN is not necessarily related to any changes in ATP-sensitive K^+^ channel conductance.

It is noteworthy that the observed increase in the whole-cell *I*_K(M)_ by NGEN did not appear to match its increase in K_M_-channel activity. The reason for this discrepancy is currently unclear. However, it is likely that the NGEN effects are much dependent on some receptors and/or intracellular second messenger systems which are possibly washed out during our whole-cell recordings. It remains to be further clarified whether any changes in receptors and/or transmembrane signal pathways are involved in the NGEN effect on K_M_ channels.

In the present study, under a cell-attached recording, we found that addition of NGEN decreased the firing frequency of spontaneous action currents in NSC-34 cells. The reduction of firing frequency caused by NGEN in these cells could be primarily explained by its activation of K_M_ channels, because linopirdine can significantly reverse NGEN-mediated inhibition of spontaneous action currents. It was also noted that when the recording pipette was filled with iberiotoxin (200 nM) or apamin (200 nM), NGEN-induced reduction in the firing of action currents still existed (data not shown). Moreover, because *I*_K(erg)_ was relatively not subject to block by NGEN at a concentration of 10 μM, lack of stimulatory effect of NGEN on the spontaneous firing of action currents could be demonstrated in the presence of this compound.

Activation of BK_Ca_ channels induced by NGEN in vascular myocytes reported previously [18] was noted to share similar characteristics shown here in HEK293T cells in which α-*hSlo* channels are functionally expressed. Our results also suggest that NGEN may bind to a site located in the cytoplasmic side of the α-subunit. In addition, BMS-204352 and meclofenamic acid, known to be activators of BK_Ca_ channels [13,37], was shown to activate K_V_7/K_M_ channels [13]. Therefore, these two types of ion channels is most likely to share the unique motifs with which some small compounds such as NGEN can interact.

Another notable issue is the subunit composition of the BK_Ca_ channels. In this study, we used homomeric α-*hSlo* channels and imply the consequences for neuronal firing. However, in the central nervous system, α subunits are accompanied by accessory β4 proteins [38]. β4 subunits were described to change channel pharmacology and current phenotype dramatically [38,39], and even produce profound effect on the neuronal firing [40]. Therefore, it is important to investigate to what extent NGEN produces any effects on α-*hSlo* + β4 channels, although the results showing that homomeric α-*hSlo* channels are activated by this compound are valuable from mechanistic standpoints.

There is evidence to show that NGEN is a target for K_M_ channel. In our study, we also found that this compound could be a direct stimulator of BK_Ca_ channels. With the aid of Blastx program (http://blast.ncbi.nlm.nih.gov/), we further examined the similarity of amino acid sequence between the α-subunit of BK_Ca_ channel (KCNMA1) and KCNQ3 protein. Interestingly, a portion of BK_Ca_-channel α-subunit (AAI4497.1), to which the sequence of KCNQ3 (AAI28577.1) shares the similarity (26%), is located at 182-381. This region are noted to correspond with the ion transport domain of the BK_Ca_-channel α-subunit. Therefore, it is tempting to speculate that NGEN or other structurally similar compounds can interact at this region to influence the activity of BK_Ca_ channels or/and K_V_7-encoded channels.

In the modeled hippocampal neurons presented herein, burst firing of APs in response to brief current injection was readily generated and an interaction of two slow variables could be derived. In other words, as shown in Figure 10, a slow autocatalytic variable (**n**) correspond to the activation gate of T-type Ca^2+^ channel, while a slow negative feedback variable (**o**) is the activation gate of K_Ca_ channel. In this system, the results from fast-slow analysis led us to suggest that the subtle interaction of activation gates between T-type Ca^2+^ channel and K_Ca_ channel is able to generate slow oscillations that move the fast subsystem from a stable non-oscillatory regime into a repetitive spiking regime and back again. Superimposition of the voltage nullcline (Figure 10) computed with the gates (**o** and **n**) designated as parameters is able to illustrate where this bifurcation emerges. It is tempting to speculate that the presence of NGEN potentially alters the firing behaviors of central neurons in vivo, assuming that those neurons functionally express the activity of both K_M_ and BK_Ca_ channels.

## Conclusions

The stimulatory effect of NGEN on native *I*_K(M)_ expressed in central neurons could have an impact on the functioning of the neurons. Further work is required to see whether the activity of K_M_ channels stimulated by this compound is responsible for the antinociceptive or antioxidative actions of different compounds known to activate *I*_K(M)_. Besides that, NGEN-induced actions on the stimulation of K_M_ and BK_Ca_ channels may combine to affect the functional activities if both channels are expressed in central neurons in vivo. Our findings raise the possibility that NGEN-mediated changes in ion currents of neurons described above are closely linked to its possible neuroprotective actions [41].

## Methods

### Drugs and solutions

Naringenin (NGEN; 4',5,7-trihydroxyflavanone) was obtained from MP Biomedicals (Solon, OH). Linopirdine, flupirdine, meclofenamic acid and tetrodotoxin were obtained from Sigma-Aldrich (St. Louis, MO), apamin, iberiotoxin and verruculogen were from Alomone Labs (Jerusalem, Israel), and gabazine and glibenclamide were from Tocris Cookson (Bristol, UK). Azimilide was a gift from Procter and Gamble Pharmaceuticals (Cincinnati, OH). All culture media, fetal bovine serum, L-glutamine, trypsin/EDTA and penicillin-streptomycin were obtained from Invitrogen (Carlsbad, CA). All other chemicals were obtained from regular commercial chemicals and of reagent grade.

The composition of the bath solution (i.e., normal Tyrode’s solution) was 136.5 mM NaCl, 5.4 mM KCl, 1.8 mM CaCl_2_, 0.53 mM MgCl_2_, 5.5 mM glucose, and 5.5 mM HEPES-NaOH buffer, pH 7.4. To record *I*_K(Ca)_ or *I*_K(M)_, the recording pipettes were backfilled with a solution consisting of 130 mM K-aspartate, 20 mM KCl, 1 mM KH_2_PO_4_, 1 mM MgCl_2_, 3 mM Na_2_ATP, 0.1 mM Na_2_GTP, 0.1 mM EGTA, and 5 mM HEPES-KOH buffer, pH 7.2. To measure *I*_K(erg)_, cells were bathed in a high-K^+^, Ca^2+^-free solution containing 130 mM KCl, 10 mM NaCl, 3 mM MgCl_2_, 6 mM glucose, and 10 mM HEPES-KOH buffer, pH 7.4. For single-channel recordings, high K^+^ bathing solution contained 145 mM KCl, 0.53 mM MgCl_2_, and 5 mM HEPES-KOH buffer, pH 7.4, and pipette solution contained 145 mM KCl, 2 mM MgCl_2_, and 5 mM HEPES-KOH buffer, pH 7.2. The value of free Ca^2+^ concentration was calculated based on the method from http://www.stanford.edu/~cpatton/downloads.htm.

### Cell preparations

NSC-34 neuronal cells were originally produced by fusion of motor neuron-enriched, embryonic mouse spinal cords with mouse neuroblastoma [24,25]. They were routinely maintained in 1:1 mixture of DMEM and Ham’s F12 medium supplemented with 10% (v/v) FBS and 1% penicillin-streptomycin. Cultures were incubated at 37°C in a humidified environment of 5% CO_2_/95% air. The medium was often replenished every 2-3 days for removal of non-adhering cells. To slow cell proliferation and enhance their maturation towards a differentiated state, before confluence, cells were grown in 1:1 DMEM plus Ham’s F12 medium supplemented with 1% FBS. To observe neurite growth in these cells, a Nikon Eclipse Ti-E inverted microscope (Li Trading Co., Taipei, Taiwan) equipped with a five-megapixel cooled digital camera was commonly used. The camera was connected to a personal computer controlled by NIS-Element BR3.0 software (Nikon; Kanagawa, Japan).

HEK293T cell line was obtained from the American Type Culture Collection (CRL-11268; Manassas, VA). Cells were grown in DMEM supplemented with 10% FBS, 2 mM L-glutamine and 1% penicillin-streptomycin at 37°C in an atmosphere of 5% CO_2_ and 95% air incubator. For the transfection of HEK293T cells, cells at a number of 0.8-2.4 × 10^5^ were seeded on the 35-mm culture plate for 24 hours.

### Transfection

The pCMV6-XL4 vector containing human BK_Ca_-channel pore forming α-subunit cDNA (α-*hSlo*; NM_002247.1) was obtained from Origene Technologies (Rockville, MD). The α-*hSlo* gene is recognized to encode a functional BK_Ca_ channel. The expression plasmid was transfected into HEK293T cells for transient expression [31,42]. In brief, the expression plasmid was prepared in 150 mM NaCl as a diluent solution. PEI (ExGen 500; MBI Fermentas, Hanover, MD) and plasmid were mixed together and incubated for 10 min at room temperature for adequate binding of the plasmid to PEI. Plasmid-PEI mixture solution was subsequently added to the 24-well plate and centrifuged at 280 *g* for 5 min. After centrifugation, transfected cells were incubated at 37°C for additional 48 hours. The expression of α-*hSlo* channels was determined by either immunofluorescence staining or electrophysiological measurements.

### Electrophysiological measurements

NSC-34 or HEK293T cells used for electrophysiological experiments were dissociated and an aliquot of cell suspension was subsequently transferred to a recording chamber mounted on the stage of an inverted fluorescent microscope (CKX-41; Olympus, Tokyo, Japan). The cells were bathed at room temperature (20-25°C) in normal Tyrode’s solution containing 1.8 mM CaCl_2_. Patch pipettes were made from Kimax-51 glass capillaries (Kimble; Vineland, NJ) using a PP-830 electrode puller (Narishige, Tokyo, Japan) or a P-97 Flaming/Brown micropipette puller (Sutter; Novato, CA), and their tips were then fire-polished with an MF-83 microforge (Narishige). The pipettes used had a resistance of 3-5 MΩ when immersed in different solutions as described above. Patch-clamp recordings were made in cell-attached, inside-out or whole-cell configuration by means of an RK-400 (Bio-Logic, Claix, France) or an Axopatch 200B patch-clamp amplifier (Molecular Devices; Sunnyvale, CA) [25,29].

Action currents that reflect APs were measured from NSC-34 neuronal cells by means of cell-attached voltage-clamp recordings as described previously [29,30]. The potential was held at the level of the resting potential (~-70 mV). Action current measurements were used to allow quantification of the underlying AP frequency under the condition where the intracellular contents remain unchanged. The waveform in action current appearing as a brief spike in the downward direction was mainly due to the capacitive current, which was shaped as the first derivative of the AP.

### Data analyses

To calculate the percentage increase of NGEN on *I*_K(M)_, each cell was depolarized from -20 to -50 mV, the amplitude of *I*_K(M)_ measured at the end of hyperpolarizing pulse in the presence of 300 μM NGEN was taken to be 100%. The NGEN concentration required to increase 50% of current amplitude was determined using a Hill function, $y=\frac{E_{\max}\times {\left[C\right]}^{n_H}}{E{C}_{50}+{\left[C\right]}^{n_H}},$ where [C] is the NGEN concentration, EC_50_ and n_H_ are the concentration required for a 50% increase and the Hill coefficient, respectively; and *E*_max_ is the NGEN-induced maximal increase in the amplitude of *I*_K(M)_ (i.e., linopirdine-sensitive current).

The relationships between the membrane potentials and the *I*_K(M)_ conductance obtained before and after the addition of NGEN (10 μM) were fitted with a Boltzmann function of the following form: $\frac{G}{G_{\max }}=\frac{1}{1+ \exp \left[\frac{-\left(V-{V}_{1/2}\right)qF}{RT}\right]},$ where G_max_ is the maximal conductance of *I*_K(M)_, *V*_1/2_ is the voltage at which there is half-maximal activation, *q* is the apparent gating charge, *F* is Faraday’s constant, *R* is the universal gas constant, and *T* is the absolute temperature. The solver subroutine built in Microsoft Excel (Redmond, WA) was commonly used to fit the data by a least-squares minimization procedure.

Single-channel currents of BK_Ca_ or K_M_ channels were analyzed using pCLAMP 9.2 software (Molecular Devices). Multigaussian adjustments of the amplitude distributions among channels were used to determine single-channel currents. The channel open probabilities were generally evaluated using an iterative process to minimize the χ^2^ calculated when an adequate number of independent observations were made. For dwell time analyses, only one single channel in the patch was used. The single-channel conductance was determined by a linear regression using the mean values of current amplitudes measured at different levels of holding potentials.

Data points presented here represent the means ± SEM. Values of n indicate the number of cells from which the data were obtained. The paired or unpaired Student’s *t* test and one-way analysis of variance with a least-significance difference method for multiple comparisons were used for the statistical evaluation of difference among means. Statistical analyses were performed using SPSS 14.0 (SPSS Inc., Chicago, IL). Since we intended to make assertions about the variability of means that could be collected from a random cohort derived from the population concerned, we believe that the standard error shown in this study could be more appropriate than the standard deviation. The level of statistical significance was set at *p* < 0.05.

### Computer simulations

To evaluate how changes in *I*_K(M)_ and/or *I*_K(Ca)_ influence neuronal firing, a theoretical model with bursting firing of APs was adapted from the work of Xu and Clancy [27]. This model is primarily based on the electrophysiological properties of hippocampal CA3 pyramidal neurons and it comprises the delayed-rectifier K^+^ current, the transient K^+^ current, the fast Na^+^ current, the T-, L- and N-type Ca^2+^ current, Ca^2+^-activated K^+^ current, and after-hyperpolarizing K^+^ current. The Markov model of Na^+^ (i.e., SCN1A) channel was incorporated to the modeled neuron [27]. In the present simulations, the conductance values and reversal potentials used to solve the set of differential equations are listed in Table 1.

The model neuron used in this work generally behaves according to the modified Hodgkin-Huxley scheme, except that the formulations of Na current was based on a Markov model. The equation for *V* is given by $Cm\frac{dV}{dt}=-\left({I}_{Na}+{I}_{CaT}+{I}_{CaL}+{I}_{CaN}+{I}_{KDR}+{I}_A+{I}_{KCa}+{I}_{AHP}+{I}_{Leak}\right)+{I}_{app},$ where *V* denotes the membrane potential; C_m_ is the membrane capacitance; *I*_Na_ is the fast Na^+^ current; *I*_*CaT*_, *I*_CaL_, and *I*_CaN_ are the T-, L- and N-type Ca^2+^ currents, respectively; *I*_KDR_ is the delayed-rectifier K^+^ current; *I*_A_ is the transient K^+^ current; *I*_KCa_ is the Ca^2+^-activated K^+^ current; *I*_AHP_ is the afterhyperpolarization current; *I*_Leak_ is the leak current; and *I*_app_ is the injected current.

Computer programs shown in this study were either written in C++ programming language or came with the XPP simulation package available at http://www.math.pitt.edu/~bard/xpp/xpp.html [33]. Parts of numerical simulations were validated in Microsoft Excel. They were generally run under a Hewlett Packard xw9300 workstation (Palo Alto, CA). The ordinary differential equations were solved numerically using the explicit Euler method with a time step of 0.001 msec.

## Abbreviations

AP: Action potential
BK_Ca_: Large-conductance Ca^2+^-activated K^+^
DMEM: Dulbecco's modified Eagle's medium
FBS: Fetal bovine serum
g_KCa_: Ca^2+^-activated K^+^ current conductance
g_M_: M-type K^+^ current conductance
HEK: Human embryonic kidney
HERG: Human *ether-à-go-go* related gene
*hSlo*: Human slowpoke
*I-V* relation: The current-voltage relation
*I*_K(Ca)_: Ca^2+^-activated K^+^ current
*I*_K(erg)_: *Ether-à-go-go* related gene-mediated K^+^ current
*I*_K(M)_: M-type K^+^ current
K_Ca_: Ca^2+^-activated K^+^
K_M_: M-type K^+^
K_Na_: Na^+^-activated K^+^
K_V_: Voltage-gated K^+^
NGEN: Naringenin
PEI: Polyethylenimine
